# Effect of transcranial magnetic stimulation on focal hand dystonia: a systematic review and meta-analysis

**DOI:** 10.3389/dyst.2026.16666

**Published:** 2026-08-13

**Authors:** Zeynep B. Simsek, Patrick J. Mulcahey, Michael W. Lutz, Angel V. Peterchev, Noreen Bukhari-Parlakturk

**Affiliations:** 1 Department of Neurology, Duke University School of Medicine, Durham, NC, United States,; 2 Medical Scientist Training Program, Duke University School of Medicine, Durham, NC, United States,; 3 Department of Psychiatry, and Behavioral Sciences, Duke University School of Medicine, Durham, NC, United States,; 4 Department of Electrical and Computer Engineering, Duke University, Durham, NC, United States,; 5 Department of Neurosurgery, Duke University, Durham, NC, United States,; 6 Department of Biomedical Engineering, Duke University, Durham, NC, United States,; 7 Duke Institute for Brain Sciences, Duke University, Durham, NC, United States

**Keywords:** focal hand dystonia, meta-analysis, noninvasive brain stimulation, systematic review, transcranial magnetic stimulation

## Abstract

**Background:**

Focal hand dystonia (FHD) is a rare and disabling movement disorder with limited treatment options. Transcranial magnetic stimulation (TMS) has shown promise as a non-invasive neuromodulation therapy for dystonia, but its efficacy in FHD remains unclear due to heterogeneity across prior studies and mixed results from broader noninvasive brain stimulation meta-analyses.

**Objective:**

To systematically review and synthesize the effects of TMS on behavioral symptoms in individuals with FHD.

**Methods:**

A systematic review and meta-analysis was conducted in accordance with PRISMA guidelines. PubMed, Embase, and Web of Science were searched through January 2026 for studies applying TMS in individuals with FHD. Studies reporting behavioral outcome measures of dystonia severity were included. Random-effects meta-analyses were performed to estimate pooled effect sizes using Hedges’ g for (1) pre-versus post-TMS comparisons and (2) active-TMS versus sham-TMS comparisons. Study quality and risk of bias were assessed using Cochrane Risk of Bias and PEDro scales. Publication bias was assessed using funnel plot and Egger’s test.

**Results:**

Nine studies met inclusion criteria, comprising a total of 111 individuals with FHD. Meta-analysis demonstrated a moderate and statistically significant improvement in dystonia symptoms following TMS compared with pre-TMS (Hedges’ g = 0.36, 95% CI [0.17, 0.56], p < 0.001). In studies including sham controls (n = 8; 102 participants), active-TMS was associated with significantly greater symptom improvement compared with sham stimulation (Hedges’ g = 0.35, 95% CI [0.12, 0.57], p = 0.002). Between study heterogeneity was low for both analyses, and no evidence of significant publication bias was detected.

**Conclusion:**

TMS is associated with a mild to moderate improvement in behavioral symptoms of FHD. These findings support further investigation of TMS as a non-invasive therapeutic option for FHD and highlight the need for larger, well-designed clinical trials to optimize stimulation parameters and assess durability of treatment effects.

## Introduction

Focal hand dystonia (FHD) is a permanently disabling neurological disorder characterized by involuntary muscle contractions and abnormal postures that impair fine motor control of the hand [1]. FHD patients are most commonly affected in skilled hand use such as writing, typing, and playing musical instruments. FHD frequently presents as an isolated syndrome in the adulthood and can contribute to an abrupt end to professional careers [2]. FHD is rare, with an estimated prevalence of 15 per million persons [3].

There are currently no disease modifying treatments for FHD. Symptomatic treatments such as botulinum toxin injections and oral medications offer limited and temporary relief [4]. Deep brain stimulation (DBS) is an invasive brain surgery that is a treatment option for generalized and cervical dystonia but not FHD [4]. As a result, the treatment landscape for FHD is poor and novel treatment options are urgently needed.

Transcranial magnetic stimulation (TMS) is a non-invasive brain stimulation technique that has shown early promise as a treatment for FHD [5]. TMS involves an electromagnetic coil placed on the scalp to excite or inhibit neurons noninvasively [6]. TMS is an FDA approved treatment for depression, obsessive compulsive disorder, smoking cessation, and migraine [7–10]. TMS has also been studied as a treatment for dystonia [5]. A prior meta-analysis of 27 studies applying all types of noninvasive brain stimulation technologies (TMS, transcranial direct current stimulation, transcranial alternating current stimulation) in multiple dystonia subtypes (FHD, cervical dystonia, lower limb dystonia, acquired dystonias) showed significant benefit in improvement of behavioral symptoms [11]. However, the effect size was small (random-effects Hedges’ g = 0.21, p = 0.002) with an unclear risk of bias assessment, significant between study heterogeneity (I^2^ = 45.04%, and p = 0.012) and a trend towards publication bias (p = 0.06) [11]. Given the unclear interpretation of this larger meta-analysis, it is not yet clear if TMS is a potentially effective treatment for FHD.

Therefore, the primary aim of this systematic review and meta-analysis was to pool only studies applying TMS in FHD and evaluate its effect on behavioral symptoms of dystonia. Due to a limited number of clinical trials applying TMS in FHD, subgroup analyses of stimulation protocols or target sites were not performed. This FHD specific review will examine the evidence for repurposing an already FDA-approved treatment as a potential option for FHD patients.

## Methods

### Search strategy

This review was conducted in accordance with the Preferred Reporting Items for Systematic Reviews and Meta-Analysis (PRISMA) statement, which is displayed in Figure 1. Web of Science, Pubmed, and Embase were searched in January 2026 using a combination of the following terms: “transcranial magnetic stimulation” AND dystonia”, OR “focal hand dystonia”, OR “musician’s dystonia”, OR “writer’s cramp.” No publication status or year limiters were applied; however, only studies reported in English were considered. The reference lists of all included articles were searched for studies missed in the initial search. All studies resulting from the searches were uploaded to Covidence software. As this study did not involve any interaction with human subjects, no ethical approval or consent was obtained.

### Eligibility criteria

Studies were screened using inclusion and exclusion criteria based on the PICO (participants, intervention, control, outcome) framework. Studies were first selected for qualitative review based on the following criteria: (P) participants who had a clinical diagnosis of FHD, with a study sample size of 3 or more; (I) TMS used as an intervention intended to reduce dystonia symptom severity; (C) no comparison group or randomization necessary; and (O) a behavioral outcome measure that assessed changes in clinical symptoms of dystonia [12]. Studies were selected for quantitative review (i.e., any statistical analysis) if (C) included a comparison group of FHD controls who received sham stimulation (parallel trials), or a design where FHD patients received both sham and real stimulation (crossover trials); (O) as above.

### Data collection and data extraction

Literature search results were exported to Covidence. Two reviewers (ZBS and PJM) independently screened titles and abstracts obtained from the literature search against the inclusion and exclusion criteria. Full-text articles were then assessed against inclusion criteria, with disagreements resolved through discussion, and where necessary by a third member of the study team (NBP). Following the screening and inclusion of full text articles, data were extracted from individual studies into Covidence spreadsheets and exported into Microsoft Excel spreadsheets, including participant demographics, clinical information, clinical trial design, TMS stimulation site, TMS stimulation parameters and behavioral outcome measures. The primary outcome measures were changes in dystonic behavior post-TMS compared to pre-TMS, or active-TMS compared to sham-TMS. Dystonic behavior included digital behavioral measures, clinician rating scales and patient reported outcomes. In this review, we defined digital behavioral measures as behavior measured using digital tools such as measures of tracking error, and pen pressure generated from a digital writing assay. Clinician rating scales included Burke Fahn Marsden Dystonia Severity scale and patient reported outcomes included Arms Dystonia Disability scale [13, 14]. All three categories of measures were extracted and included in the meta-analyses. In studies where graphs or images were used to report the means and standard deviations (SDs) standard errors (SE), Plot Digitizer (version 3.0; https://plotdigitizer.com) software was used to extract values. Plot Digitizer software has high interrater reliability and is more accurate than traditional methods for extracting data from figures and graphs [15, 16].

### Study quality assessment

Two independent reviewers (ZBS and PJM) assessed the methodological quality of the included studies using Cochrane Collaboration’s Risk of Bias (RoB) checklists [17]. The RoB checklist assesses studies on the domain’s randomization, allocation concealment, blinding of participants and personnel, eligibility criteria, intention to treat, between group comparisons, point estimates and variability, and adequate followups defined as greater than 1 week after intervention [17]. Each domain was judged to be of low, unclear, or high risk of bias, with an overall judgment given for each study, of low (low risk of bias for all domains), unclear (some concerns in at least one domain), or high (high risk of bias in at least one domain) risk [17]. The methodological quality of each study was also assessed using the PEDro scale [18]. A higher PEDro score indicates a higher quality of the trial, with scores of 9 and 10 indicating excellent quality, 6–8 indicating good quality, 4 and 5 indicating fair quality, and less than 4 indicating poor quality [18].

### Effect size estimation

Due to the small sample sizes of the included articles, a Hedges’ g effect size was calculated to correct for potential overestimation of the population standardized mean difference (SMD) [19]. For all studies, a Hedges’ g and SD was first calculated for each outcome measure [19]. For studies with more than one outcome measure, the mean and SD of each measure before and after active-TMS and a mean and SD before and after sham-TMS were extracted and used to calculate a mean difference and SD for active-TMS and a mean difference and SD for sham-TMS. For each measure, the mean difference was then used to calculate a Hedge’s g and SE for active-TMS and a Hedge’s g and SE for sham-TMS. The Hedge’s g and SE were then averaged across measures to generate a Hedge’s g and SE for active-TMS and sham-TMS for each study. The direction of the outcome measure was adjusted so that positive values indicated improved dystonia behavioral outcomes, and negative values indicated worsened dystonia behavioral outcomes. The mean Hedge’s g for each TMS condition in each study was then used to calculate two pooled effect sizes: 1-change in dystonic behavior before and after TMS (pre-TMS vs. post-TMS) and 2-change in dystonia behavior after the two TMS conditions (active-TMS vs. sham-TMS). The pooled effect sizes were calculated using a random-effects model in Comprehensive Meta-Analysis (CMA; version 4) software. Both study level and the overall pooled effect size were considered significant if p < 0.05. All meta-analysis forest plots and funnel plots were prepared in CMA software. Between-study heterogeneity in effect sizes was quantified using the Q-test and I^2^ statistic [20, 21]. The Q-statistic provides a test of the null hypothesis that all studies in the analysis share a common effect size [20]. If all studies shared the same true effect size, the expected value of Q would be equal to the degrees of freedom (the number of studies minus 1). The I^2^ statistic for heterogeneity is measured as low, moderate, or high for I^2^ values of 25%, 50%, and 75%, respectively [21].

### Publication bias

The presence of publication bias across studies was assessed using funnel plots where effect sizes for each study were plotted against their standard error [19]. In the absence of publication bias, symmetrical distribution of effect sizes around the overall effect size is observed, indicating randomness due to a sampling error [19]. Egger’s test quantitatively evaluates the significance of any detected asymmetry, with non-significant asymmetry suggesting no publication bias [19]. The statistical significance level was set at 0.1 because the statistical power of the publication bias tests is generally low.

## Results

### Study selection

The electronic search strategy yielded 330 papers, including 84 duplicates removed automatically by the Covidence software and 45 removed manually (Figure 1). After screening the titles and abstracts for PICO framework, 201 full-text publications were retrieved and screened for eligibility. Of these, nine articles met the inclusion criteria and were included in the qualitative and quantitative synthesis (Table 1). Although three of the nine clinical trials originated from the same hospital and investigator, the study characteristics of the three articles were different [24, 27, 28]. The three studies, therefore, were deemed to be independent and included in the quantitative analysis [24, 27, 28].

### Clinical trial design

Six of the nine included studies used a crossover (full or partial) study design while the remaining three used a parallel study design (Table 1). The sample sizes of the included studies ranged from 6 to 18 participants. All studies delivered two interventions. For eight of the nine studies, the two interventions were active-TMS and sham-TMS. One study delivered the same active-TMS combined with sensorimotor retraining (SMR) or control therapy.

### Participant characteristics

A total of 111 individuals with FHD who received TMS treatment were included in the meta-analysis comparing pre-TMS and post-TMS (Figure 2) and 102 patients with FHD were included in the meta-analysis comparing active-TMS and sham-TMS (Figure 3). The mean age and sex distribution across the 111 FHD participants was 45.5 [SD 4.98] years with 63% male and 37% female. The duration of FHD symptoms ranged from 5.7 years to 16.4 years (Table 1). Overall, the FHD study cohort across the nine clinical trials represented a diverse patient cohort.

### TMS stimulation site

Six of the nine studies targeted TMS to the premotor cortex (PMC) [22–24, 26–28, 30]. Across the nine studies, TMS was also targeted to the motor cortex (MC, two studies), primary somatosensory cortex (S1, two studies), supplementary motor area (SMA, one study), and ipsilateral cerebellum (one study) [29]. Of note, cortical target for TMS was identified using scalp measurements in seven of the nine studies. The remaining two studies used fMRI to identify the cortical target for TMS delivery [25, 30].

### TMS stimulation parameters

TMS stimulation parameters were categorized by frequency of stimulation, intensity, waveform, and duration of each TMS session. Since some studies delivered multiple TMS sessions over five consecutive days, the total pulses was also reported for comparison. The majority of studies (6/9) delivered low-frequency rTMS (1 Hz or 0.2 Hz), while two studies delivered continuous theta-burst stimulation (cTBS) and one study used high-frequency rTMS (10 Hz) (Table 1). The intensity of TMS stimulation was in the range of 80%–90% of resting motor threshold (RMT) in half of the studies (5/9) and 80%–100% of active motor threshold (AMT) in the other half. The waveform was biphasic in most of the studies (7/9) and monophasic in the rest. The duration of each TMS session ranged from 40 s for cTBS to 40 min for 10 Hz rTMS. Of note, the 40-min rTMS session consisted of 5 min of TMS delivery interleaved with 5 min of motor behavior for four repetitions. The motor behavior was used to prime the motor circuitry prior to TMS delivery. Across all nine studies, the 40-min study was the only one that delivered TMS after a motor priming task [30]. The remaining studies delivered TMS during rest state. The total number of pulses delivered per stimulation site ranged from 600 to 9,000. All studies used figure-of-eight coils to deliver TMS.

### Outcome measures and durability of effect size

To assess TMS effect in FHD, all nine clinical trials collected dystonic behavioral measures including digital behavioral measures, clinician rating scales and patient reported outcomes (Table 4). A majority of the digital behavioral measures included kinematic features of writing such as pen pressure, pen velocity and peak accelerations during writing. The durability of the effect size ranged from immediately after TMS delivery to 28 days post intervention (Table 1).

### Study quality assessment

Methodological quality of studies was assessed using two different scaling systems: risk of bias scaling (Table 2) and physiotherapy evidence database (PEDro) scale (Table 3). Using the risk of bias scale, all nine studies showed low risk of bias in randomization, eligibility criteria, between group comparisons, and reporting of point estimates and measures of variability. A majority (5/9) of the studies also reported blinding. In these cases, blinding was reported primarily at the level of study subjects. A limited number of studies (3/9) also reported blinding of study assessors and only one study reported blinding of the TMS technician. A majority of the studies (6/9) were designed and maintained an intention to treat analysis. Adequate follow up (> 1 week after each TMS session) was observed in a majority (6/9) of studies. For the remaining studies (2/9), only one TMS session was delivered and follow up was performed on the same day. The PEDro scale was used to assess the overall study quality (Table 3). All nine studies were at minimum fair quality with a majority (7/9) meeting criteria for good quality.

### Effect size estimation and measures of heterogeneity

Across all nine studies, a meta-analysis was performed comparing dystonic behavior before and after TMS (Table 4). A mild-moderate significant effect size favoring post-TMS and an overall reduction in dystonic behavior was observed with the random effects Hedges’ g = 0.36, 95% CI [0.17, 0.56], p < 0.001 (Figure 2). Between study heterogeneity as measured by Cochrane’s Q (X2) was 2.24 with 8 degrees of freedom. Since the Cochrane’s Q was less than the degrees of freedom, the amount of between-study variance in the observed effects was actually less than we would expect to see based on sampling error alone. Therefore, the variance of true effects was estimated as zero, and all indices of heterogeneity (I^2^, tau^2^ and tau) were set to zero.

Since a pre-post meta-analysis is vulnerable to regression to the mean, placebo effects, learning effects, and natural fluctuations in dystonic behavior, a second meta-analysis was performed to compare dystonic behavior after sham-TMS and active-TMS (Figure 3). As one study did not include a sham-TMS group, it was excluded from this analysis and the remaining eight studies were used to calculate a pooled effect size. The second meta-analysis also showed a moderate effect size favoring active-TMS for an overall reduction in dystonia with the random effects Hedges’ g = 0.35, 95% CI [0.12, 0.57], p = 0.002 (Figure 3). Between-study heterogeneity as measured by Cochrane’s Q (X2) was 0.93 with 7 degrees of freedom. Since the Cochrane’s Q was less than the degrees of freedom, the amount of between-study variance in the observed effects was less than we would expect to see based on sampling error alone. Therefore, the variance of true effects was estimated as zero, and all indices of heterogeneity (I^2^, tau^2^, and tau) were set to zero.

### Publication bias

Since studies that report a treatment effect are more likely to be published and therefore lead to an overestimate of the true treatment effect, it is important to assess the likely extent of publication bias and its potential impact on the true TMS effect in dystonia. A funnel plot was, therefore, used to measure publication bias. In the absence of publication bias, we would expect the published studies to be distributed symmetrically around the combined effect size with larger study sizes clustering near the top of the funnel plot. A funnel plot analysis of the nine studies comparing pre-TMS vs. post-TMS (Figure 4A) and sham-TMS vs. active-TMS (Figure 4B) demonstrated a symmetrical distribution. The effect size calculated with the imputed studies (black diamond) was the same as the effect size with the observed studies (open diamond) suggesting that the extent of publication bias was low.

An Egger’s test was also performed for both meta-analyses. For pre-TMS vs. post-TMS meta-analysis, the Egger’s test indicated no significant publication bias (2.09, 95% CI [−0.95, 5.13], p = 0.148). Similarly, for sham-TMS vs. active TMS, the Egger’s test also indicated no significant publication bias (−1.05, 95% CI [−4.16, 207], p = 0.443). Overall, using a funnel plot and Egger’s test, we found no evidence of publication bias overestimating the TMS effect size in dystonia.

However, funnel plots and Egger’s test have limited power when fewer than ten studies are included, so the absence of detected publication bias should be interpreted with caution.

## Discussion

The primary aim of this systematic review and meta-analysis was to assess the effect of TMS in FHD. Overall, the two meta-analyses of eight to nine studies demonstrated a mild-moderate and significant effect of TMS in improving symptoms of FHD. Furthermore, the risk of study bias was found to be low with a majority of the studies demonstrating good quality. The between-study heterogeneity was also found to be low, and the risk of publication bias did not significantly impact the estimated TMS treatment effect. Therefore, the mild-moderate effect of TMS in improving behavioral symptoms in FHD are likely to be the true predicted effect.

Demonstration that TMS can have a mild to moderate effect size in improving behavioral symptoms across 111 FHD participants is encouraging given the rarity of the disorder and the paucity of effective treatments. In comparison, DBS was approved as a clinical treatment for generalized dystonia through a humanitarian device exemption pathway for rare disorders. The data supporting DBS approval was retrospectively based on symptom improvement in 40 patients across three clinical studies [31–33]. In contrast, the present meta-analysis demonstrates mild to moderate improvement in a larger cohort of patients using a non-invasive approach. The present meta-analysis, therefore, is providing evidence for the potential of TMS as a non-invasive clinical therapy option for FHD patients.

A systematic review of the nine studies also highlights the urgent need to fund more clinical trials investigating the optimal TMS parameters for clinical therapy in FHD. The TMS parameter space is vast that a systematic investigation of all possible iterations is not possible nor advisable. Instead, a majority of the nine studies in this meta-analysis demonstrated behavioral benefit after TMS was delivered to the premotor cortex with low (1 Hz or 0.2 Hz) or “inhibitory” (cTBS) frequency. In all of these studies, TMS was delivered while participants were in a resting brain state. Therefore, low frequency TMS to the premotor cortex during a naïve resting brain state may be a viable clinical treatment option to consider investigating in a larger scale clinical trial.

Our group also demonstrated that high frequency (10 Hz) TMS to the primary somatosensory cortex during a non-naïve resting state (after a motor priming task) may also allow for symptom improvement [30]. Overall, the dystonic behavioral improvement after TMS using two different stimulation paradigms leads us to speculate that there may be more than one set of TMS parameters that will benefit FHD patients. It is the interplay between brain target site, stimulation protocol (frequency, intensity, waveform) and brain state (naïve vs. non-naïve) that ultimately will determine the extent of benefits patient will receive and the optimal parameters that should be approved as a clinical treatment. Mechanistic studies further exploring this interplay are needed to develop a deeper understanding of TMS effect in dystonia.

Although the present study is focused on TMS in FHD, TMS may also be a promising therapy for other dystonia subtypes. However, it is important to emphasize that dystonia is a clinical syndrome and not a disease [1]. There is a growing body of evidence that each subtype of adult focal dystonia (cervical dystonia, blepharospasm, laryngeal dystonia) may have a different brain mechanism [34]. Specifically, there may be common brain regions that contribute to an overall abnormality in the motor network but the extent of involvement of each brain region may vary by the dystonia subtype [34]. Therefore, the optimal TMS parameters (target site, stimulation protocol and brain state) will need to be empirically tested for each dystonia subtype. To ensure good clinical trial design, it is highly recommended that each dystonia subtype be considered as a unique group and tested individually or in a basket trial design. A comparison of the same TMS stimulation parameters across multiple dystonia subtypes will also advance our understanding of the similarity and differences in brain mechanism across the dystonia subtypes.

### Study limitation

This review has several limitations that should be considered. First, the limited number of clinical trials investigating the effects of TMS in FHD precluded a comprehensive meta-analysis including secondary analysis of moderators of TMS benefit. This gap in the literature may be addressed in the future as more rigorous and larger scale clinical trials of TMS in FHD and other dystonia subtypes are funded. Second, the TMS studies in FHD in this meta-analysis also varied in brain targets, TMS parameters, TMS dosing, and brain state during TMS delivery. The finding of very low heterogeneity may reflect low sampling power to detect heterogeneity rather than true homogeneity. Third, the included clinical trials provided limited data on long-term TMS effects, with few studies extending follow-up beyond 1 month. This lack of long-term findings hampered our ability to draw conclusions regarding the prolonged effects of TMS on dystonia subtypes. Lastly, there were no uniform digital behavioral outcome measures used by all clinical trials which likely led to the variability in evaluating TMS effect on dystonia symptoms. Nevertheless, the behavioral measures used across the nine trials were reported in sufficient detail to allow calculation of a pooled effect and a low between study heterogeneity.

## Conclusion

This systematic review and meta-analysis showed that TMS is a promising approach to improve dystonia motor symptoms in individuals with FHD. TMS to the premotor cortex or primary somatosensory cortex may allow for behavioral improvement in FHD. More clinical trials systematically comparing TMS parameters in FHD are needed to advance this promising technique towards clinical therapy. Overall, the findings of the present meta-analysis encourage further larger and carefully designed clinical trials to assess the potential clinical value of TMS in FHD.

## Figures and Tables

**FIGURE 1:**
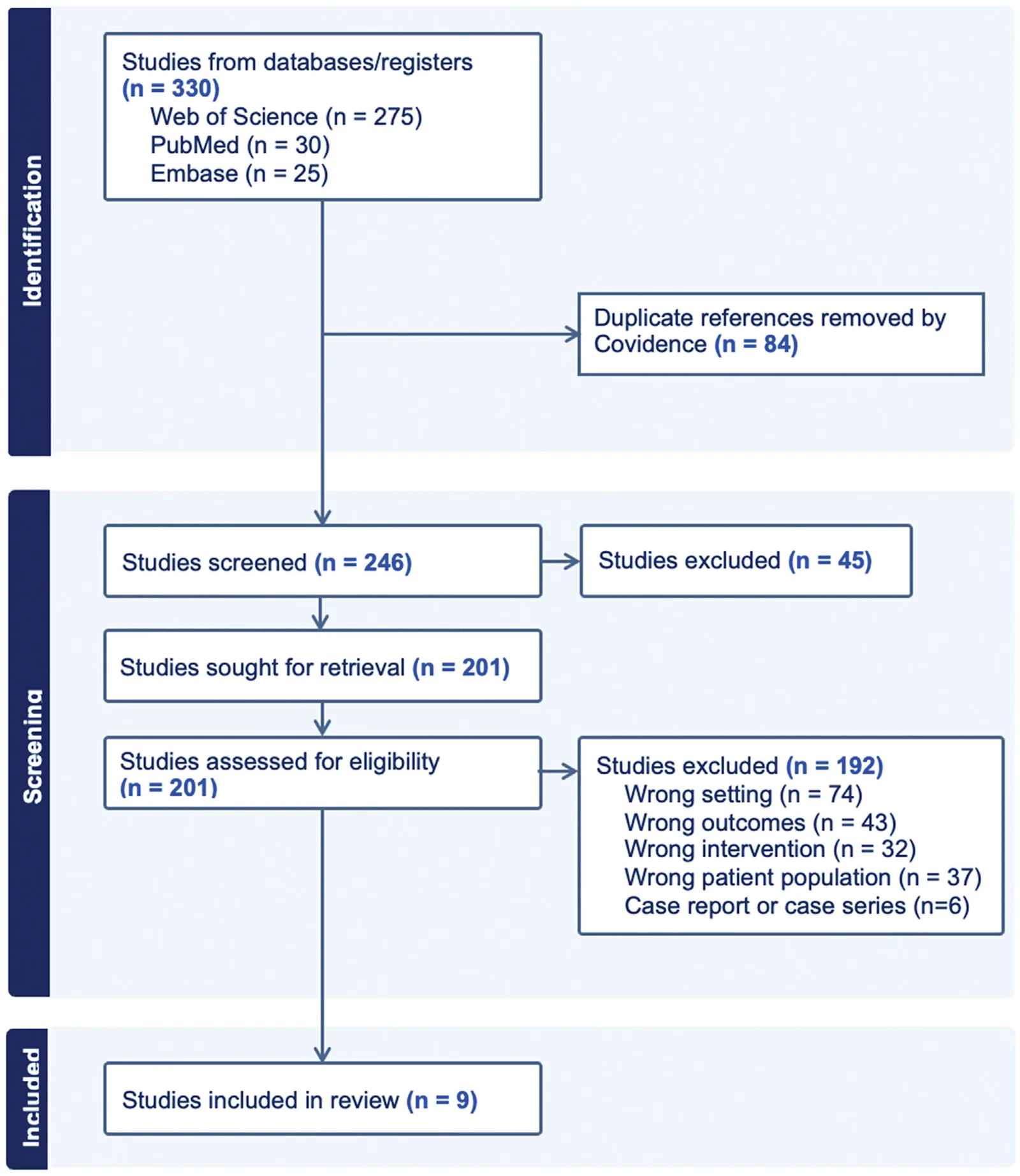
PRISMA flowchart of study selection. 246 studies were identified in the screening process of which 201 were reviewed and 9 studies were deemed eligible for quantitative synthesis.

**FIGURE 2:**
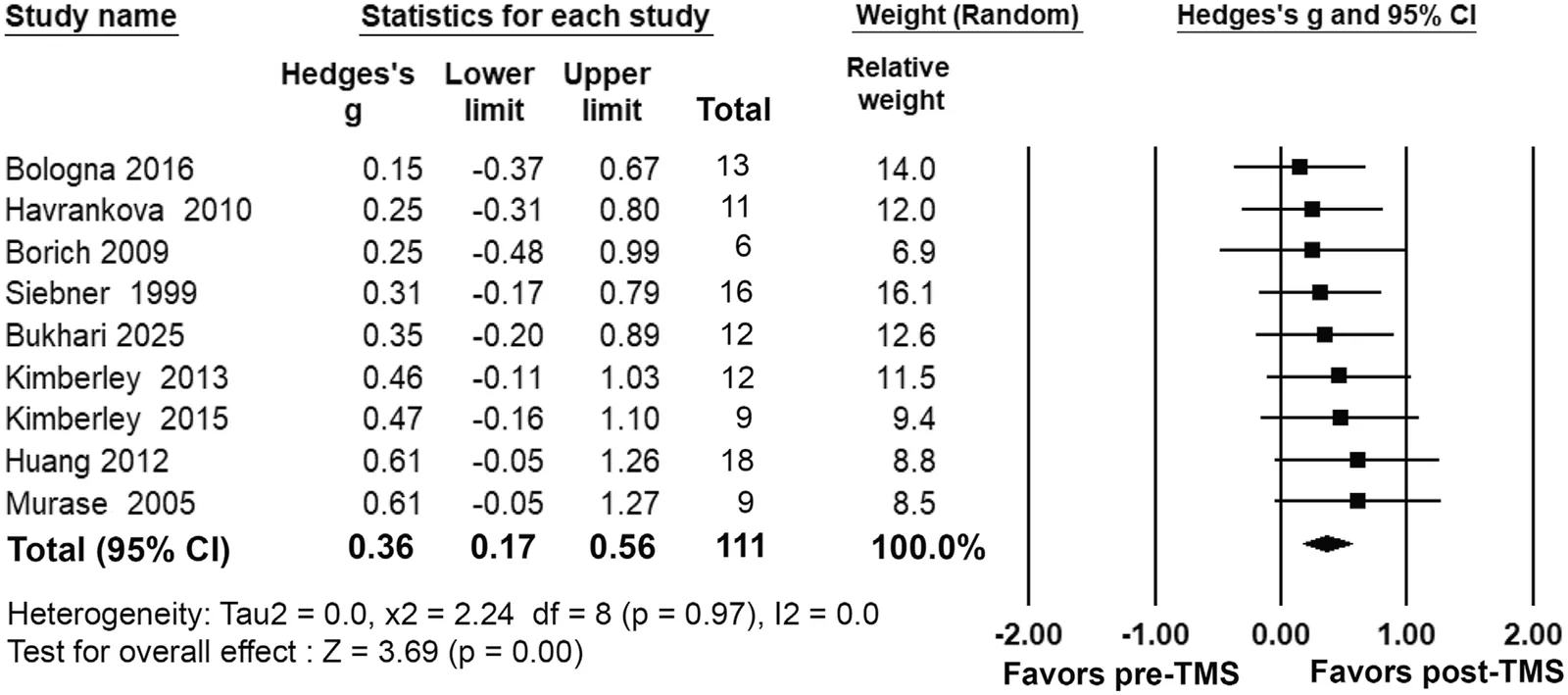
Forest plots comparing dystonic behavior before and after TMS across nine clinical trials in FHD patients.

**FIGURE 3:**
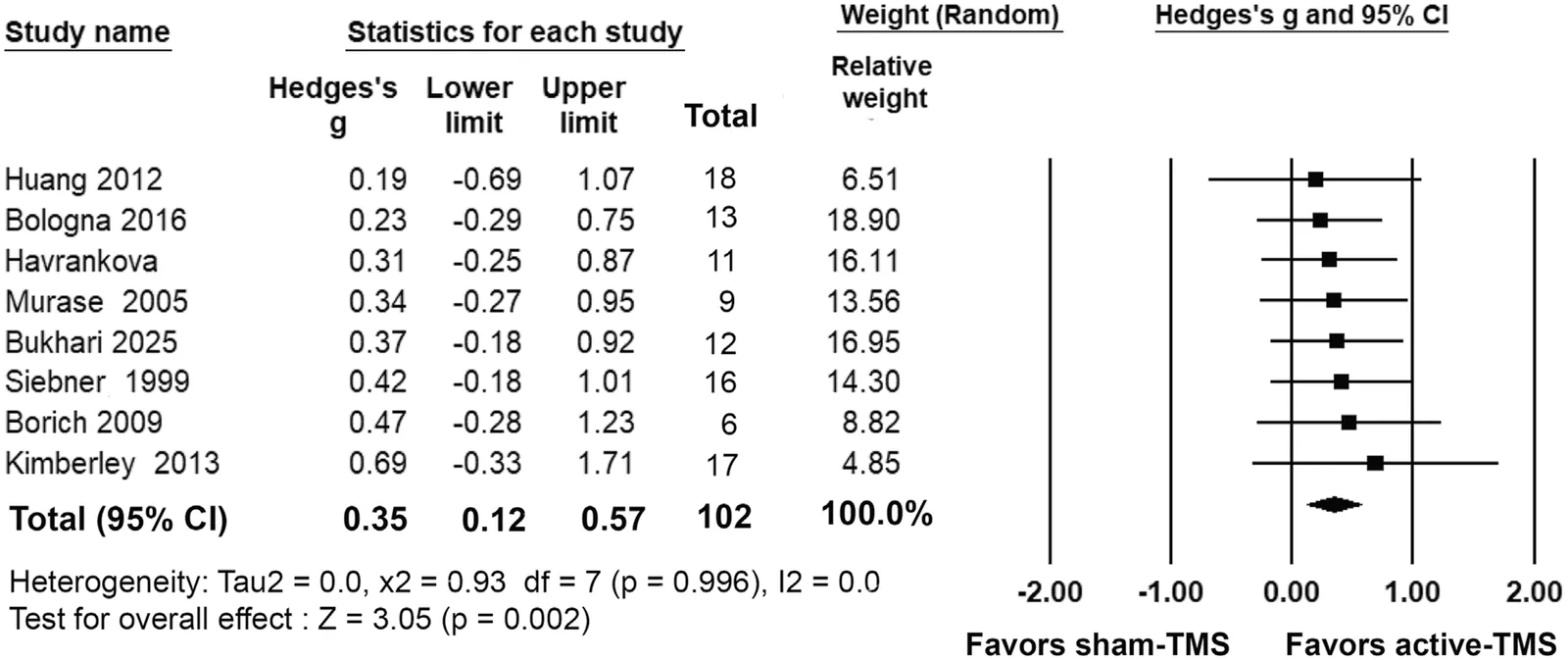
Forest plots comparing the change in dystonic behavior after sham-TMS and active-TMS across eight clinical trials of FHD patients.

**FIGURE 4:**
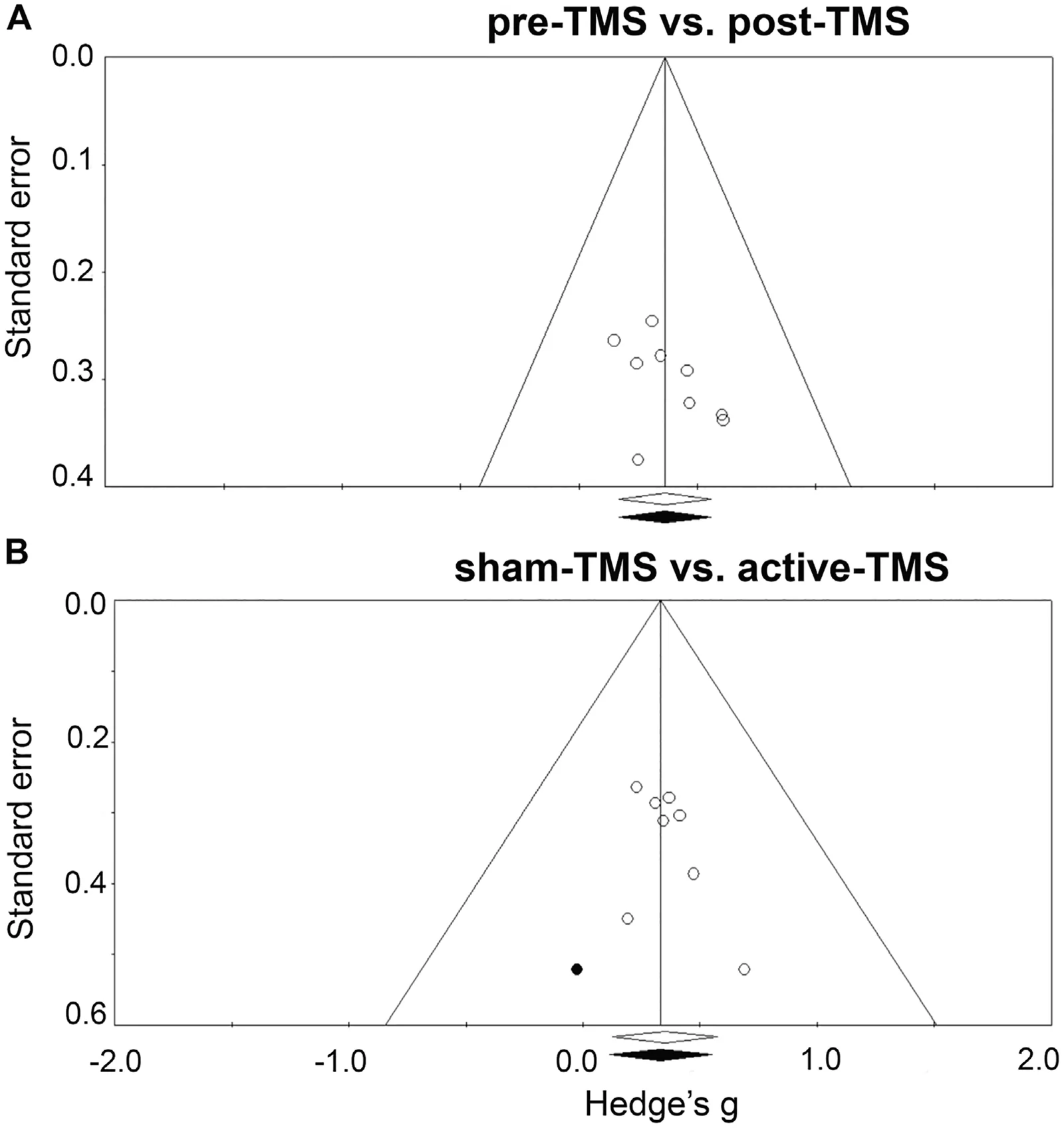
Funnel plots evaluating the risk of publication bias when comparing (A) pre-TMS vs. post-TMS and (B) sham-TMS vs. active-TMS. Open circles represent observed studies, and closed circles represent imputed studies. Open diamond represents observed effect size and closed diamond represents effect size after combining observed and imputed studies. Overall, the observed effect size is unchanged by imputed studies. Therefore, the extent of publication bias for both comparisons is low and does not affect findings of this meta-analysis.

**TABLE 1:** Study characteristics.

Study	Clinical trial		Participants		TMS stimulation			Behavioral measures	Follow-up duration
	Design	Arms	FHD	Healthy	Site	Parameters	Total pulses		
Siebner [22]	Partial crossover	Active rTMS vs. sham	n = 16 Mean age = 47 years Sex = 6 F/10 M Mean disease duration = 10 years	n = 11 40 years 4 F/7 M	Left MC	Freq: 1 Hz Intensity: 90% RMT Waveform: biphasic Duration: 30 min	1800	Writing pressure, stroke frequency, writing velocity, inversions in velocity per stroke	Immediately
Murase [23]	Crossover	Active rTMS vs. sham	n = 9 38 years 3 F/6 M 7.7 years	n = 7 36 years 2 F/5 M	MC, PMC, SMA	Freq: 0.2 Hz Intensity: 85% AMT for MC and PMC, 100% AMT for SMA Waveform: monophasic Duration: 20 min	750 (250 per site)	Tracking error, pen pressure	Immediately
Borich [24]	Partial crossover	Active rTMS vs. sham	n = 6 46.5 years 1 F/5 M 9.8 years	n = 9 33 years 3 F/6 M	Contralateral PMC	Freq: 1 Hz Intensity: 90% RMT Waveform: monophasic Duration: 15 min	4,500 (900/day, 5 days)	Pen error, pen pressure, pen velocity	15 days
Havrankova [25]	Crossover	Active rTMS vs. sham	n = 11 40.3 years 8 F/3 M 5.7 years		Contralateral S1	Freq: 1 Hz Intensity: 90% AMT, Waveform: biphasic Duration: 30 min	9,000 (1800/day, 5 days)	2M-writing test, BFMDS, function of the hand, pain intensity	28 days
Huang [26]	Parallel	Active TBS- TMS vs. sham	n = 18 42.1 years 8 F/10 M 11 years	n = 8 42 years 5 F/3 M	Left PMC	Freq: cTBS (3 pulses of 50 Hz repeated at 5 Hz) Intensity: 80% AMT active TMS, 60% AMT coil flipped over sham Waveform: biphasic Duration: 40 s	3,000 (600/day, 5 days)	Writing speed, Gibson spiral maze test	5 days
Kimberley [27]	Parallel	Active rTMS + SMR vs. Active rTMS + control	n = 17 46.5 years 7 F/10 M 9 years		Contralateral dPMC	Freq: 1 Hz Intensity: 90% RMT Waveform: biphasic Duration: 30 min	9,000 (1800/day, 5 days)	Axial pen force, change in velocity	15 days
Kimberley [28]	Crossover	Active rTMS vs. sham	n = 9 46 years 3 F/6 M 9 years		PMC	Freq: 1 Hz Intensity: 80% RMT Waveform: biphasic Duration: 20 min	1,200	Sentence jerk, sentence pressure, sentence velocity, ADDS, WCRS movement score, WCRS writing speed	5 days
Bologna [29]	Parallel	Active TBS- TMS vs. sham	n = 13 48.5 years 6 F/9 M 6.2 years	n = 13 49.9 years 6 F/9 M	Ipsilateral cerebellum	Freq: cTBS (3 pulses of 50 Hz repeated at 5 Hz) Intensity: 80% AMT Waveform: biphasic Duration: 40 s	600	Duration, velocity peak, acceleration peak, straightness, smoothness, overshooting	45 min
Bukhari-Parlakturk [30]	Crossover	Active rTMS vs. sham	n = 12 55 years 1 F/11 M 16.4 years		PMC, S1	Freq: 10 Hz Intensity: 90% RMT Waveform: biphasic Duration: 40 min	8,000 (4,000 per site)	Peak accelerations	60–90 min

RCT, Randomized Controlled Trial; FHD, Focal Hand Dystonia; HC, Healthy Control; F, Female; M, Male; SD, Standard Deviation; MC, Motor Cortex; PMC, Premotor Cortex; dPMC, dorsal Premotor Cortex; SMA, Supplementary Motor Area; S1, Somatosensory Cortex; rTMS, Repetitive Transcranial Magnetic Stimulation; cTBS, Continuous Theta Burst Stimulation; SMR, sensorimotor training; Hz, Hertz; RMT, Resting Motor Threshold; AMT, Active Motor Threshold; 2MWT, 2-Minute Writing Test; BFMDS, Burke-Fahn-Marsden Dystonia Scale; ADDS, Arm Dystonia Disability Scale; WCRS, Writer’s Cramp Rating Scale.

**TABLE 2:** Risk of bias assessment for individual studies.

Study ID	Randomization	Allocation concealment	Blinding			Eligibility criteria	Intention to treat	Between group comparisons	Point estimates and variability	Adequate follow-ups
			Subjects	Assessors	TMS tech					
Siebner [22]	Low	Low	Low	High	High	Low	Low	Low	Low	High
Murase [23]	Low	Low	Low	High	High	Low	Low	Low	Low	High
Borich [24]	Low	Low	Low	High	High	Low	Low	Low	Low	Low
Havrankova [25]	Low	Low	Low	Low	High	Low	Low	Low	Low	Low
Huang [26]	Low	High	High	High	High	Low	High	Low	Low	Low
Kimberley [27]	Low	High	High	High	High	Low	High	Low	Low	Low
Kimberley [28]	Low	Unclear	High	Low	High	Low	Low	Low	Low	Low
Bologna [29]	Low	Unclear	High	High	High	Low	Low	Low	Low	Low
Bukhari-Parlakturk [30]	Low	Low	Low	Low	Low	Low	Low	Low	Low	High

**TABLE 3:** Physiotherapy evidence database (PEDro) score list.

Study ID	Eligibility criteria	Randomization	Allocation concealment	Blinding			Intention to treat	Between group comparisons	Point estimates and variability	Adequate follow-ups	Total
				Subjects	Assessors	TMS tech					
Siebner [22]	Yes	1	1	1	0	0	1	1	1	0	6
Murase [23]	Yes	1	1	1	0	0	1	1	1	0	6
Borich [24]	Yes	1	1	1	0	0	1	1	1	1	7
Havrankova [25]	Yes	1	1	1	1	0	1	1	1	1	8
Huang [26]	Yes	1	0	0	0	0	0	1	1	1	4
Kimberley [27]	Yes	1	0	0	0	0	0	1	1	1	4
Kimberley [28]	Yes	1	1	0	1	0	1	1	1	1	7
Bologna [29]	Yes	1	1	0	0	0	1	1	1	1	6
Bukhari-Parlakturk [30]	Yes	1	1	1	1	1	1	1	1	0	8

A higher PEDro, score indicates a higher quality of the trial. Scores 9–10 = excellent quality, 6–8 = good quality, 4–5 = fair quality, and <4 = poor quality.

**TABLE 4:** Effect size estimations of dystonic behavioral measures.

Study	Measures	Active TMS					Sham TMS				
		Pre-TMS	Post-TMS	Change score	Hedge’s g	SEM	Pre-TMS	Post-TMS	Change score	Hedge’s g	SEM
		Mean (SD)	Mean (SD)	Mean difference (SD)			Mean (SD)	Mean (SD)	Mean difference (SD)		
Siebner [22]	Writing velocity (mm/s)	36.70 (58.40)	39.50 (68.80)	2.80 (64.23)	0.04	0.24	-	-	-	-	-
	Vertical writing pressure (N)	2.57 (0.70)	2.27 (0.67)	0.30 (0.69)	0.42	0.25	-	-	-	-	-
	Writing pressure (N)	2.58 (0.70)	2.24 (0.66)	0.34 (0.68)	0.47	0.25	2.58 (0.70)	2.56 (0.75)	0.02 (0.73)	0.42	0.30
Murase [23]	Tracking error (cm)	0.14 (0.03)	0.12 (0.02)	0.03 (0.03)	0.85	0.36	0.14 (0.06)	0.14 (0.04)	0.01 (0.05)	0.35	0.31
	Pen pressure (N)	98.70 (37.90)	84.80 (26.00)	13.90 (33.57)	0.37	0.31	82.40 (33.10)	81.10 (34.00)	1.30 (33.56)	0.34	0.31
Borich [24]	Tracing error (a.u.)	12.02 (0.32)	11.77 (0.59)	0.25 (0.51)	0.35	0.38	11.87 (0.59)	12.19 (0.34)	−0.32 (0.51)	0.63	0.42
	Pen pressure (g)	146.27 (28.51)	136.40 (27.77)	9.87 (28.15)	0.26	0.37	161.72 (21.70)	128.54 (20.17)	33.18 (20.98)	0.53	0.39
	Pen velocity (m/s)	2.70 (0.53)	2.81 (0.53)	0.11 (0.53)	0.15	0.37	2.43 (0.02)	2.79 (0.56)	0.36 (0.55)	0.26	0.35
Havrankova [25]	2M-writing test (n)	170.00 (68.00)	189.00 (73.00)	19.00 (70.63)	0.25	0.28	178.00 (73.00)	174.00 (71.00)	−4.00 (72.02)	0.30	0.29
	BFMDS	3.00 (1.20)	2.50 (0.50)	0.50 (1.04)	0.44	0.29	2.80 (1.20)	2.70 (1.20)	0.10 (1.20)	0.33	0.29
	Pain intensity (0–10 scale)	2.90 (3.00)	2.10 (3.00)	0.80 (3.00)	0.25	0.28	2.60 (3.00)	2.90 (3.00)	−0.30 (3.00)	0.34	0.29
	Hand function (0–10 scale)	3.70 (2.00)	3.80 (2.00)	0.10 (2.00)	0.05	0.28	4.10 (2.00)	3.60 (2.00)	−0.50 (2.00)	0.28	0.28
Huang [26]	Writing speed (n)	64.02 (12.21)	75.50 (17.70)	11.48 (15.69)	0.66	0.34	71.43 (12.40)	80.20 (16.05)	8.77 (14.5)	0.17	0.45
	Spiral maze test (n)	35.16 (10.02)	29.30 (9.08)	−5.86 (9.59)	0.55	0.33	31.61 (7.85)	27.70 (7.41)	−3.91 (7.64)	0.21	0.45
Kimberley [27]	Axial pen force (N)	-	-	0.21 (0.24)	0.79	0.31	-	-	−0.02 (0.14)	0.98	0.53
	Change in velocity (m/s)	-	-	−0.16 (1.18)	0.13	0.27	-	-	−0.60 (0.47)	0.40	0.51
Bologna [29]	Duration (ms)	485.44 (91.26)	482.11 (84.69)	3.33 (88.16)	0.04	0.26	506.69 (111.20)	492.76 (93.24)	13.93 (103.40)	0.10	0.26
	Velocity peak (m/s)	1.42 (0.32)	1.41 (0.36)	0.01 (0.34)	0.03	0.26	1.32 (0.29)	1.41 (0.32)	−0.09 (0.31)	0.29	0.27
	Acceleration peak (m/s^2^)	8.95 (2.81)	9.02 (3.17)	−0.07 (3.01)	0.02	0.26	7.97 (2.63)	8.84 (3.24)	−0.87 (2.98)	0.25	0.26
	Straightness (%)	105.16 (2.27)	104.08 (2.02)	1.08 (2.16)	0.47	0.28	104.85 (2.20)	104.41 (1.98)	0.44 (2.10)	0.28	0.27
	Smoothness (n)	1.30 (0.61)	1.12 (0.25)	0.18 (0.53)	0.32	0.27	1.61 (0.97)	1.15 (0.22)	0.46 (0.89)	0.34	0.27
	Overshooting (mm)	7.62 (4.87)	7.45 (5.16)	0.17 (5.02)	0.03	0.26	5.48 (3.35)	5.88 (3.50)	−0.40 (3.43)	0.12	0.26
Bukhari-Parlakturk [30]	Peak accelerations (n) - PSC	350.49 (29.06)	338.28 (35.49)	12.21 (32.75)	0.35	0.28	343.61 (28.75)	343.41 (24.41)	0.20 (26.84)	0.37	0.28

Study	Measures	Active-TMS					
		Lower CI	Upper CI	p-value	Mean difference	Hedge’s g	SEM
Kimberley [28]	Sentence jerk (n)	−0.43	0.16	0.26	0.14	0.33	0.31
	Sentence pressure (N)	−0.46	0.16	0.58	0.15	0.34	0.31
	Sentence velocity (m/s)	−5.34	18.71	0.21	−6.68	0.39	0.31
	ADDS (0–100 scale)	0	0.25	0.03	0.13	0.72	0.35
	WCRS movement (0–28 score)	−0.61	7.27	0.16	3.33	0.59	0.33
	WCRS writing speed (0–2 score)	−0.12	0.56	0.05	0.22	0.45	0.32

ms, milliseconds; m/s, meters per second; %, percent; n, number or counts; mm, millimeters; N, Normal.
